# Editorial: Multidisciplinary management of cancer patients with immune-related adverse events from checkpoint inhibitors

**DOI:** 10.3389/fmed.2022.1104382

**Published:** 2023-01-05

**Authors:** Mar Riveiro-Barciela, Enriqueta Felip, María E. Suarez-Almazor

**Affiliations:** ^1^Liver Unit, Internal Medicine Department, Hospital Universitari Vall d'Hebron, Vall d'Hebron Barcelona Hospital Campus, Barcelona, Spain; ^2^Centro de Investigación Biomédica en Red de Enfermedades Hepáticas y Digestivas (CIBERehd), Instituto de Salud Carlos III, Madrid, Spain; ^3^Department of Medicine, Universitat Autònoma de Barcelona (UAB), Barcelona, Spain; ^4^Medical Oncology Department, Vall d'Hebron University Hospital, Vall d'Hebron Institute of Oncology, Barcelona, Spain; ^5^Department of Health Services Research and Section of Rheumatology and Clinical Immunology, The University of Texas MD Anderson Cancer Center, Houston, TX, United States

**Keywords:** immunotherapy, toxicity, cancer, oncology—discipline, special populations

Cancer immunotherapy with immune checkpoint inhibitors (ICI) has revolutionized the management of many frequent advanced tumors, such as lung cancer (1). These therapies have markedly improved the survival of cancer patients decreasing the rates of recurrence and progression of the underlying malignancies (2, 3). However, the widespread use of ICIs has also lead to an exponential rise of immune-related adverse events (irAEs) associated with these drugs.

As summarized in Figure 1, real-world practice has brought to light substantial challenges in many aspects of the management of irAEs, for instance in the diversity of clinical manifestations, the grading of severity and strategies for treatment, with concerns about the potential effect of immunosuppressants on the efficacy of ICIs. Furthermore, immunotherapy is now used in special cancer populations such as those infected by human immunodeficiency virus (HIV), patients who have received solid-organ transplantation, individuals with underlying autoimmune disorders, or patients with latent infections such as tuberculosis o viral hepatitis, who can develop reactivation of their infections when they receive therapy with ICIs. Altogether, these issues highlight the vital importance of collaborative teamwork in order to optimize the prognosis of patients who develop irAEs or other complications as a consequence of receiving ICI. These challenges are the theme of this Research Topic of *Frontiers in Medicine* entitled “*Multidisciplinary Management of Cancer Patients with Immune-Related Adverse Events from Checkpoint Inhibitors*.”

**Figure 1:**
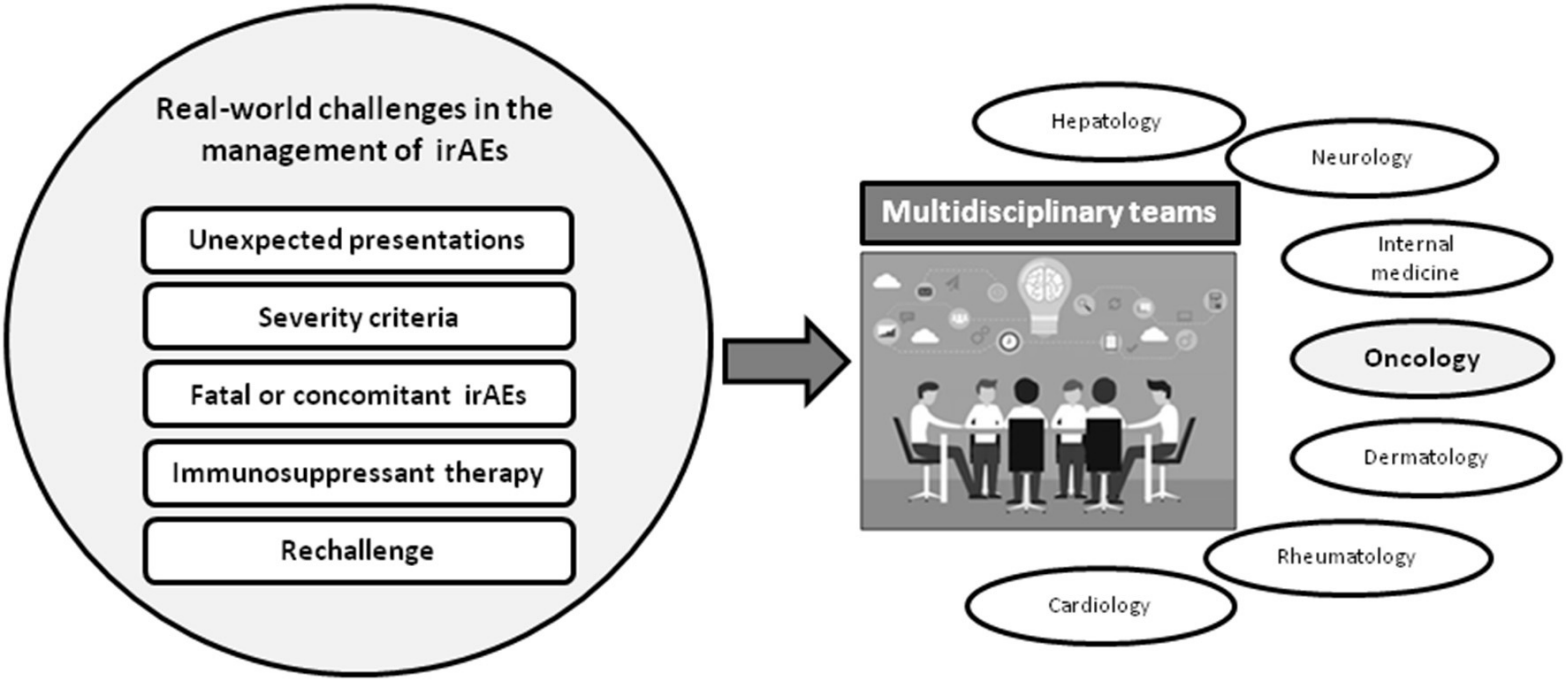
Pitfalls of real-world practice immune-related adverse events associated with immune checkpoint inhibitors (ICIs) and need of multidisciplinary teams for their management. The difficulties and differences between irAEs reported at the clinical trials and real-world practice highlight the importance of the multidisciplinary management of patients with severe irAEs.

## Immunotherapy in special populations

It is well-known that patients who have received solid-organ transplantation are at increased risk of subsequent cancer, and may be eligible to receive ICI. On this topic, Bermejo et al. summarize the outcomes and controversies surrounding the treatment with ICIs in patients with prior kidney transplantation. One of the hot topics in this field is the risk of acute rejection associated with ICI therapy. Concomitant treatment with mTOR monotherapy, low-dose corticosteroids or even a “dynamic immunosuppression” scheme seems to reduce the incidence of rejection. The efficacy of this last approach will be assessed in a prospective cohort, and robust results may be available soon (Bermejo et al.).

The increased survival of patients with HIV, currently comparable to those non-infected, highlights the need to provide access to effective therapeutic cancer therapies in this population, as shown in the paper by Aguilar-Company et al. In this setting, incidence of irAEs seems similar to that observed in the general cancer population, without changes in plasma viral loads (Aguilar-Company et al.).

The coexistence of an autoimmune disorder with cancer is of special relevance for therapy with ICIs, as there are risks of flares of the pre-existing autoimmune disease, as well as a potential for higher incidence of irAEs (Aguilar-Company et al.). As learnt from studies of patients with inflammatory bowel disease or rheumatoid arthritis, patients with good control of their autoimmune disorder prior to beginning ICI therapy are less likely to develop exacerbations during immunotherapy (4) (Aguilar-Company et al.). Fortunately, concomitant treatment with immunosuppressive drugs, including anti-TNF agents, seems safe in patients receiving ICIs (Robles-Alonso et al.).

## Immune checkpoint inhibitors and risk of latent infections

Widespread use of ICI has highlighted the risk of reactivation of latent infections such as tuberculosis or viral hepatitis with these agents.

In registry studies all patients with hepatitis B and C had to be virologically suppressed, with the exception of those with hepatocellular carcinoma. Although, ICIs have no impact on hepatitis C virus (HCV) and could even decrease HCV-RNA, screening for HCV is highly recommended in cancer patients to assess potential concomitant cirrhosis and further risk of decompensation (5). Concerning hepatitis B, patients with chronic hepatitis B, that is those with positive HBsAg, are at risk of reactivation when receiving ICIs (6), so they can benefit from concomitant antiviral prophylaxis, as is recommended for those undergoing chemotherapy (5). Hitherto, data on the risk of hepatitis B reactivation on patients with resolved infection i.e., testing negative for HBsAg but positive for anti-HBc, is scarce. In this Research Topic, Aceituno et al. reported the absence of hepatitis B reactivation in a cohort of 75 subjects with resolved HBV infection. Interestingly, authors also reported the relatively low awareness of oncologists about the risk of viral hepatitis reactivation in patients on ICIs, with only 55% of subjects having the complete serology panel performed before immunotherapy was started (Aceituno et al.).

There is a need for further studies regarding the risk-benefit and the proper strategy for management of latent tuberculosis in individuals about to initiate ICIs. Current evidence does not clearly support routine latent tuberculosis infection screening, and treatment for latent tuberculosis should be weighed on an individual basis, accounting for potential pharmacological interactions, risk of hepatotoxicity and expected survival (Aguilar-Company et al.).

## Treatment of severe immune-related adverse events

One of the hottest topics on the management of irAEs is the treatment of severe grade-3 and grade-4 adverse events. Corticosteroids have been the backbone of therapy of severe irAEs in both registry studies and international guidelines (7, 8). However, real-world practice has revealed that for many patients with severe irAEs, temporal discontinuation of ICIs may result in improvement without need of immunosuppressant therapy, for instances in immune-mediated hepatitis (9, 10). Moreover, data on some irAEs has shown the potential benefits of early access to corticosteroid-sparing agents. On this regard, studies of real-world clinical practice indicate that specific therapy for irAEs in accordance with the autoimmune disorders they mimic may be beneficial. For example, in the case of immune-related colitis, early access to endoscopy can identify subjects with ulcers or pancolitis who will benefit from infliximab therapy (4). Similarly individuals with severe immune-related arthritis could benefit from early therapy with TNF or IL-6 inhibitors (11).

However, an outstanding issue associated with the use of immunosuppressant drugs is their potential harm on cancer progression, especially taking into account the advanced stage of tumors in the majority of patients undergoing ICIs. As summarized by Bruera et al., the potential deleterious effect of corticosteroids on cancer progression seems to be associated with dose and timing of use. Concomitant treatment with corticosteroids at the initiation of ICIs can negatively impact overall and progression-free survival, whereas temporal or intermittent corticosteroids such as are used often for irAEs, do not seem to negatively impact survival (Bruera et al.).

## Rechallenge with immune checkpoint inhibitors after a severe irAE

Once an irAE is resolved, the next step is to assess the possibility of ICI rechallenge. The majority of clinical trials and, in consequence, the international guidelines, recommend against retreatment after a severe irAE. However, a few reports have suggested a strong correlation between the development of irAEs and a better response to ICIs (12); [Cardeña-Gutiérrez and López Barahona]. Moreover, for many patients there are no further alternatives for therapy beyond ICIs. Altogether, these have led to rechallenge with ICIs after recovery from a severe irAE. Real-world data from subjects with history of a severe gastrointestinal irAE have revealed that relapse is not universal, ranging from 24% among patients with prior immune-related colitis to 35% in those with previous immune-related hepatitis (13, 14). Despite this data, retreatment with ICIs after a severe irAE, as many other aspects about immunotherapy, remains a hot topic, with low agreement even among experts on their management (15).

In summary, several challenges remain in the management of severe irAEs which require collaborative multidisciplinary efforts. As clinical trials of ICIs did not include special populations such as those with pre-exiting autoimmune diseases or transplants, or patients with chronic infections, initially there were concerns about treating with ICIs cancer patients with these disorders. As new real-world evidence emerges, it is becoming increasingly clear, that cancer patients with these concomitant comorbidities can also benefit from immunotherapy and should not be denied treatment in most cases. Careful multidisciplinary management with an emphasis in controlling concomitant comorbidities can result in successful therapy in these patients, for many of whom immunotherapy will be their last therapeutic alternative.

## Author contributions

Drafting of the manuscript: MR-B. Critical revision of the manuscript for important intellectual content: EF and MS-A. All authors contributed to the article and approved the submitted version.
